# Risk factors for not proceeding to reconstructive surgery after female genital mutilation: A cohort study of 220 women in a specialized referral center

**DOI:** 10.1002/ijgo.71094

**Published:** 2026-05-28

**Authors:** Félicia Joinau‐Zoulovits, Maëlle Silvestre De Sacy, Bruno Renevier, Sarah Abramowicz

**Affiliations:** ^1^ Université Paris Cité and Université Sorbonne Paris Nord, Inserm, INRAE, Centre for Research in Epidemiology and Statistics, Obstetric Perinatal and Paediatric Life Course Epidemiology Team OPPaLE, iWISH Paris France; ^2^ Maternity Unit, AP‐HP Bichat Hospital Paris France; ^3^ Maternity Unit Centre Hospitalier André Grégoire Montreuil France

**Keywords:** clitoral reconstruction, female genital mutilation, healthcare disparities, multidisciplinary care, social vulnerability

## Abstract

**Objective:**

This study identifies factors associated with not proceeding to clitoral reconstructive surgery among women with female genital mutilation (FGM) enrolled in a specialized surgical pathway.

**Methods:**

A retrospective cohort study was conducted at a multidisciplinary referral center in Montreuil, France, between January 2021 and December 2023. The study included 220 women who expressed a wish for reconstructive surgery during their initial assessment. Factors associated with non‐progression to surgery were identified using univariable and multivariable logistic regression, supplemented by Bayesian analysis.

**Results:**

Of the 220 women, 35% did not proceed to surgery. In the multivariable model, lack of support within the care pathway (odds ratio [OR] 3.28, 95% confidence interval [CI] 1.46–7.55; *P =* 0.004) and financial vulnerability, specifically reliance on state welfare (OR 3.43, 95% CI 1.28–9.80; *P* = 0.017) or financial support from relatives/community (OR 6.18, 95% CI 1.40–28.9; *P* = 0.017), were independently associated with non‐progression. Model discrimination was good (area under the curve 0.75). Bayesian analysis confirmed these findings (*P*[direction] >99%) and suggested that memory of the FGM event also played a role (OR 1.87, 95% credible interval 0.88–4.09; *P*[direction] 94.9%).

**Conclusion:**

Access to clitoral reconstruction is primarily determined by socioeconomic stability and social support rather than clinical factors alone. These findings highlight the need for multidisciplinary pathways that integrate social and psychological accompaniment to ensure equitable access to care for vulnerable women.

**Abbreviations**
AUC: Area under the curve
CHI: Centre hospitalier intercommunal
CI: Confidence interval
CrI: Credible interval
FGM: Female genital mutilation
FGM/C: Female genital mutilation/cutting
IQR: Interquartile range
MDT: Multidisciplinary team meeting
OR: Odds ratio
WHO: World Health Organization

## 1 INTRODUCTION

Female genital mutilation (FGM), also referred to as female genital mutilation/cutting (FGM/C), is defined by the World Health Organization (WHO) as “all procedures involving partial or total removal of the external female genitalia or other injury to the female genital organs for non‐medical reasons.”^1^ More than 230 million girls and women worldwide are estimated to be living with FGM, and over 3 million girls remain at risk each year, despite international recognition of the practice as a violation of the human rights of women and children.^2^ In settings where it is practiced, FGM reflects deep‐rooted gender inequality embedded in social, economic, and political structures and is often framed as a marker of cultural identity and a rite of passage into adulthood; it may also be linked to social expectations regarding femininity, honor, and the control of women's sexuality.^3^, ^4^

Female genital mutilation is associated with both immediate and long‐term health consequences. Acute complications include severe pain, hemorrhage, infection, urinary difficulties, and, rarely, death.^1^ Longer‐term outcomes include gynecological, urological and sexual morbidities, psychological sequelae (including post‐traumatic stress disorder and anxiety), and obstetric complications such as increased risks of caesarean birth, postpartum hemorrhage, and adverse neonatal outcomes.^5^, ^6^, ^7^, ^8^, ^9^ In 2025, the WHO issued updated recommendations on the management of FGM‐related complications, emphasizing the need for comprehensive, multidisciplinary care, including psychological support (e.g., cognitive behavioral therapy where indicated), sexual‐health counseling, information/education interventions, and deinfibulation for women with type III FGM to prevent obstetric and urological complications.^1^, ^10^, ^11^

With recent population movements, FGM is now encountered in most countries, creating growing needs for trained healthcare professionals and specialized services. In France, more than 139 000 adult women are estimated to have undergone FGM.^12^ Clitoral reconstructive surgery, described by the French urologist Pierre Foldès, aims to restore a more functional anatomy through excision of scar tissue and repositioning of the clitoral body.^13^, ^14^ In specialized centers, reconstruction is embedded in a multidisciplinary pathway rather than offered as an isolated surgical procedure. This pathway begins with medico‐social, psychological, and sexological assessment, including consultations with a specialized midwife, psychologist, and sexologist and, where appropriate, participation in peer support group sessions. Surgery is then preceded by a dedicated preoperative consultation and followed by structured clinical, psychological, and sexological follow‐up. Reimbursed by the French National Health Insurance since 2004, the procedure is rarely complicated^15^ and has been associated with improvements in sexual function, dyspareunia, perceived anatomy, and broader dimensions of well‐being and body image.^16^, ^17^, ^18^

In Seine‐Saint‐Denis, where FGM is common,^19^ a specialized multidisciplinary unit was established at Montreuil Hospital in 2017. It offers tailored pathways combining medico‐psycho‐social care with or without reconstructive surgery, as well as medico‐legal support, and is currently supported through the French “Article 51” innovation framework to improve access and coordination of care. In this context, we observed that a substantial proportion of women who initially requested clitoral reconstruction did not ultimately proceed to surgery. Yet, to our knowledge, no study has specifically examined factors associated with non‐progression to clitoral reconstructive surgery within a specialized care pathway. Although a similar surgical pathway has been reported in another French center, reasons for leaving the pathway before surgery were not documented in detail.^11^

Therefore, we conducted a retrospective, monocentric cohort study in the Montreuil specialized unit (2021–2023) to identify factors associated with not proceeding to clitoral reconstructive surgery among women enrolled in the surgical pathway. Secondary objectives were to identify the key proximal determinants of non‐progression to surgery to inform targeted strategies to reduce avoidable barriers and optimize equitable, patient‐centered access to reconstructive care.

## 2 METHODS

### 2.1 Study population

We conducted a retrospective cohort study using routinely collected data from electronic medical records. The study included women with FGM who entered the reconstructive surgery pathway between January 2021 and December 2023 in the multidisciplinary FGM unit of CHI Montreuil, a general hospital in the Île‐de‐France region, France. This unit is one of the main national referral centers for FGM care and provides integrated medical, psychological, sexological, social, and reconstructive care. Information was retrieved from electronic patient records that were filled out during each visit. The study complied with French CNIL‐MR004 and GDPR data protection regulations. All participants were informed and did not object to the reuse of their data in this study. The study was accepted by the CEROG committee under the reference #2024‐GYN‐0707.^20^

All eligible women included in this study were over 18 years of age and had expressed a wish to undergo reconstructive surgery at the initial assessment and had attended more than one consultation with members of the multidisciplinary team. Women who did not express a wish to undergo surgery, for whom the multidisciplinary team (MDT) meeting did not confirm a surgical indication, or who attended solely to obtain a medico‐legal certificate, were not included. Women were classified into two groups according to their observed pathway outcome during follow‐up: those who proceeded to clitoral reconstructive surgery and those who did not proceed to surgery after initial engagement in the pathway. This classification was based on electronic medical records and surgical scheduling records. Women were not assigned to groups by the investigators; the groups reflected the real‐life progression or non‐progression of patients through the care pathway. The care pathway includes an initial assessment with a midwife and systematic consultations with the unit's sexologist, psychologist, and gynecologist. At the end of this assessment, each case is discussed at an MDT meeting to confirm surgical eligibility and indication.

### 2.2 Handling of missing data

Missing data were handled using a predefined strategy. For descriptive analyses, missing values were reported separately for each variable and percentages were calculated among non‐missing observations. For regression analyses, univariable models were performed using all available observations for each variable (available‐case analysis). Multivariable analyses were conducted using complete case data restricted to participants with no missing values for the variables included in the final model. Given the exploratory nature of the study and the relatively limited sample size, no imputation procedures were performed.

### 2.3 Statistical analysis

Continuous variables were summarized using the median and interquartile range and categorical variables using counts and percentages. Between‐group comparisons were performed using the Wilcoxon–Mann–Whitney test for continuous variables and Pearson's *χ* ^2^‐test for categorical variables (or Fisher's exact test where appropriate). Statistical significance was defined as a two‐sided *P*‐value <0.05. All estimates are presented with their 95% confidence intervals (CIs).

Univariable binary logistic regression models were fitted for each candidate predictor, with discontinuation of the surgical pathway as the dependent variable. Crude odds ratios (ORs) with 95% CIs were reported. Variables with a *P*‐value <0.05 in univariable analyses were considered for inclusion in the multivariable model, in line with commonly used approaches in clinical epidemiology.

Variables with sparse cell counts (*n* < 5 in any category) were not included in the multivariable logistic regression models, as such data can lead to quasi‐complete separation, non‐convergence, and unreliable (potentially infinite) maximum‐likelihood estimates. Therefore, these variables were reported in univariable analyses only and interpreted with caution. The final multivariable model was constructed using candidate variables identified from the univariable screening step. Collinearity among candidate predictors was assessed using the variance inflation factor, and only variables without evidence of substantial collinearity were retained in the final model. To explore potential selection bias related to missing data, characteristics of participants included in the multivariable analysis were compared with those excluded because of missing values.

In addition to the frequentist approach, Bayesian logistic regression was performed using weakly informative priors as recommended by Gelman (normal priors centered on zero with a standard deviation of 2.5). This approach is well suited to modest sample sizes and allows estimation of directional posterior probabilities (i.e., the probability that each coefficient is positive or negative), providing an intuitive quantification of uncertainty beyond dichotomous significance testing. Eight Markov chains with 4000 iterations each were run to allow convergence toward the target posterior distribution and to obtain a sufficient number of effective samples for precise estimation of posterior quantities.

Model discrimination was evaluated using the area under the receiver operating characteristic curve (AUC), with 95% CIs computed using DeLong's method. The optimal classification threshold was identified using Youden's index.

All analyses were conducted using R version 4.5.2 (R Foundation for Statistical Computing) via RStudio. The R package gtsummary (version 2.5.0) was used to generate summary statistics, and Bayesian logistic regression analyses were performed using the *rstanarm* package.

## 3 RESULTS

Among 220 women, 142 (65%) proceeded to clitoral reconstruction and 78 (35%) did not (Table 1). Overall, the population reflected the communities usually cared for in this referral center: most women were born in Sub‐Saharan Africa (86%), mainly Mali, Côte d'Ivoire, Guinea, and Senegal, and the most frequently reported ethnic groups were Soninke, Peul, Bambara, and Malinke. Women who did not proceed were less likely to report support within the care pathway (52% vs. 73%; *P* = 0.006) and more often had recent immigration (<12 months) (13% vs. 2%; *P* = 0.002). Indicators of socioeconomic vulnerability were also more frequent among women who did not proceed, including unemployment (73% vs. 46%; *P* < 0.001), lack of health insurance (14% vs. 4%; *P* < 0.003), and reliance on state welfare benefits (53% vs. 23%; *P* < 0.001) or informal support from relatives/community (14% vs. 6%) (*P* < 0.001). Dyspareunia was more frequently reported (81% vs. 64%; *P* = 0.019), memory of the FGM event was more often reported among women who did not proceed to surgery (56% vs. 41%; *P* = 0.047), and women were younger at presentation (median 29.5 vs. 33.0 years; *P* = 0.039).

**TABLE 1:** Characteristics of the population.

	*N*	Overall (*N* = 220)^a^	Proceeded to surgery (*N* = 142)^a^	Did not proceed to surgery (*N* = 78)^a^	*P*‐value^b^
*Support for the care pathway*	195				0.006
Yes		130 (67%)	98 (73%)	32 (52%)	
Non		65 (33%)	36 (27%)	29 (48%)	
Missing data		25	8	17	
*Place of birth*	217				0.597
Sub‐Saharan Africa		187 (86%)	120 (86%)	67 (86%)	
France		27 (12%)	18 (13%)	9 (12%)	
Autre		3 (2%)	1 (1%)	2 (2%)	
Missing data		3	3	0	
*Country of origin*	216				0.241
Mali		72 (33%)	48 (35%)	24 (31%)	
Côte d'Ivoire		50 (23%)	30 (22%)	20 (26%)	
Senegal		33 (15%)	25 (18%)	8 (10%)	
Guinee		37 (17%)	19 (14%)	18 (24%)	
Other		24 (12%)	17 (11%)	7 (9%)	
Missing data		4	3	1	
*Ethnicity*	189				0.656
Bambara		32 (17%)	22 (18%)	10 (15%)	
Soninke		51 (27%)	32 (26%)	19 (29%)	
Peul		34 (18%)	23 (19%)	11 (17%)	
Malinke		18 (10%)	9 (7%)	9 (14%)	
Other		54 (28%)	37 (30%)	17 (25%)	
Missing data		31	19	12	
*Recent immigration (<12 months)*	213				0.002
Non		200 (94%)	134 (98%)	66 (87%)	
Yes		13 (6%)	3 (2%)	10 (13%)	
Missing data		7	5	2	
*Socio‐occupational category*	213				<0.001
Employee		79 (37%)	60 (44%)	19 (25%)	
Student		16 (8%)	14 (10%)	2 (3%)	
Unemployed		118 (55%)	62 (46%)	56 (73%)	
Missing data		7	6	1	
*Health insurance coverage*	205				0.003
National Health Insurance		75 (37%)	57 (45%)	18 (23%)	
CMU		77 (37%)	45 (35%)	32 (42%)	
AME		37 (18%)	21 (16%)	16 (21%)	
None		16 (8%)	5 (4%)	11 (14%)	
Missing data		15	14	1	
*Financial resources*	207				<0.001
Income		107 (52%)	85 (64%)	22 (29%)	
State welfare benefits		71 (34%)	31 (23%)	40 (53%)	
Support from relatives/community		17 (8%)	7 (6%)	10 (14%)	
Charity		12 (6%)	9 (7%)	3 (4%)	
Missing data		13	10	3	
*Marital status*	216				0.062
Married		97 (45%)	70 (51%)	27 (35%)	
Cohabiting		69 (32%)	38 (28%)	31 (40%)	
Divorced		18 (8%)	13 (9%)	5 (6%)	
Single		32 (15%)	17 (12%)	15 (19%)	
Missing data		4	4	0	
*Parity*	220				0.890
0		37 (17%)	23 (16%)	14 (18%)	
1		42 (19%)	26 (19%)	16 (21%)	
2		35 (16%)	22 (15%)	13 (17%)	
3		50 (23%)	35 (25%)	15 (19%)	
4		32 (15%)	19 (13%)	13 (17%)	
≥5		24 (10%)	17 (12%)	7 (8%)	
*History of physical violence*	212				0.383
No		124 (58%)	83 (61%)	41 (54%)	
Yes		88 (42%)	53 (39%)	35 (46%)	
Missing data		8	6	2	
*History of sexual violence*	210				0.659
No		125 (60%)	79 (58%)	46 (62%)	
Yes		85 (40%)	57 (42%)	28 (38%)	
Missing data		10	6	4	
*Forced marriage*	213				0.269
No		153 (72%)	102 (74%)	51 (67%)	
Yes		60 (28%)	35 (26%)	25 (33%)	
Missing data		7	5	2	
*Age at FGM (years)*	210	4.0 [0.5–13.0]	4.0 [0.5–13.0]	4.5 [0.5–14.0]	0.353
Missing data		10	8	2	
*Age at FGM (category)*	220				0.251
0–1 years		92 (42%)	62 (44%)	30 (38%)	
1–6 years		45 (20%)	32 (22%)	13 (17%)	
≥6 years		83 (38%)	48 (34%)	35 (45%)	
*Memory of the FGM event*	212				0.047
No		113 (53%)	79 (59%)	34 (44%)	
Yes		99 (47%)	56 (41%)	43 (56%)	
Missing data		8	7	1	
*Type of FGM*	187				0.159
Type I		25 (13%)	19 (13%)	6 (13%)	
Type II		158 (85%)	120 (86%)	38 (83%)	
Type III		2 (1%)	2 (1%)	0 (0%)	
Type IV		2 (1%)	0 (0%)	2 (4%)	
Missing data		33	1	32	
*Chronic painful complications*	212				0.260
No		175 (83%)	109 (80%)	66 (87%)	
Yes		37 (17%)	27 (20%)	10 (13%)	
Missing data		8	6	2	
*Reported dyspareunia*	213				0.012
No		64 (30%)	50 (36%)	14 (19%)	
Yes		149 (70%)	90 (64%)	59 (81%)	
Missing data		7	2	5	
*Age at initial medical care (years)*	217	32.0 [21.0–48.0]	33.0 [21.0–52.0]	29.5 [20.0–42.0]	0.039
Missing data		3	3	0	

Abbreviations: AME, Aide médicale de l'Etat; CMU, couverture maladie universelle; FGM, female genital mutilation.

^a^ *n* (%); median [5th–95th percentile]. Missing values are reported separately as a percentage of the total sample (*n* = 220); all other percentages are calculated among non‐missing observations.

^b^ Fisher's exact test; Wilcoxon rank sum test.

In univariable logistic regression, non‐progression to reconstructive surgery was associated with social vulnerability, socioeconomic precarity, and some clinical factors (Table 2). Lack of support within the care pathway (OR 2.47, 95% CI 1.31–4.66; *P* = 0.005), recent immigration within the previous 12 months (OR 6.77, 95% CI 1.99–30.9; *P* = 0.005), and unmarried status, particularly cohabiting (OR 2.12, 95% CI 1.11–4.08; *P* = 0.024) and single women (OR 2.29, 95% CI 1.00–5.24; *P* = 0.049), were associated with higher risk of not proceeding to surgery. A similar pattern was observed for indicators of socioeconomic precarity, including unemployment (OR 2.85, 95% CI 1.54–5.45; *P* = 0.001), absence of health insurance (OR 6.97, 95% CI 2.23–24.7; *P* = 0.001), and reliance on state welfare benefits (OR 4.99, 95% CI 2.60–9.82; *P* < 0.001) or support from relatives or the community (OR 5.52, 95% CI 1.91–16.8; *P* = 0.002), compared with women reporting personal income. Among clinical factors, memory of FGM (OR 1.78, 95% CI 1.02–3.16; *P* = 0.045) and reported dyspareunia (OR 2.34, 95% CI 1.21–4.74; *P* = 0.014) were associated with higher odds of non‐progression, whereas older age at initial medical care was associated with lower odds (OR 0.96 per 1‐year increase, 95% CI 0.92–0.99; *P* = 0.017). The multivariable model included seven variables and was fitted on 180 complete cases (82% of the cohort). The proportion of women not proceeding to surgery was similar among included and excluded observations (35.5% vs. 34.8%). After adjustment, lack of support within the care pathway (OR 3.28, 95% CI 1.46–7.55; *P* = 0.004) and financial vulnerability (state welfare benefits: OR 3.43, 95% CI 1.28–9.80; *P* = 0.017; support from relatives/community: OR 6.18, 95% CI 1.40–28.9; *P* = 0.017) remained independently associated with non‐progression. These two factors showed additive effects without evidence of multiplicative interaction. The final model demonstrated good discrimination (AUC 0.75).

**TABLE 2:** Factors associated with not proceeding to reconstructive surgery. Univariable and multivariable analyses.

Variable	Univariable				Adjusted multivariable^a^			
	*N*	OR	95% CI	*P*‐value	*N*	OR	95% CI	*P*‐value
*Support for care pathway*	195				180			
Yes		—	—			—	—	
No		2.47	1.31–4.66	0.005		3.28	1.46–7.55	0.004
*Recent immigration (<12 months)*	213							
No		—	—					
Yes		6.77	1.99–30.9	0.005				
*Socio‐professional category*	213							
Employed		—	—					
Student		0.45	0.07–1.81	0.320				
Unemployed		2.85	1.54–5.45	0.001				
*Health insurance coverage*	205				180			
National Health Insurance		—	—			—	—	
CMU		2.25	1.13–4.59	0.023		0.76	0.27–2.03	0.586
AME		2.41	1.04–5.63	0.040		0.62	0.17–2.03	0.439
None		6.97	2.23–24.7	0.001		1.16	0.24–5.79	0.857
*Financial resources*	207				180			
Income		—	—			—	—	
State welfare benefits		4.99	2.60–9.82	<0.001		3.43	1.28–9.80	0.017
Support from relatives/community		5.52	1.91–16.8	0.002		6.18	1.40–28.9	0.017
Charity		1.29	0.27–4.75	0.721		0.72	0.08–4.67	0.739
*Marital status*	216				180			
Married		—	—			—	—	
Cohabiting		2.12	1.11–4.08	0.024		1.66	0.69–3.98	0.256
Divorced		1.00	0.30–2.93	0.996		0.43	0.09–1.76	0.262
Single		2.29	1.00–5.24	0.049		0.62	0.18–2.01	0.435
*Memory of FGM*	212				180			
No		—	—			—	—	
Yes		1.78	1.02–3.16	0.045		1.83	0.87–3.94	0.115
*Reported dyspareunia*	213				180			
No		—	—			—	—	
Yes		2.34	1.21–4.74	0.014		1.11	0.46–2.75	0.819
*Age at initial medical care* ^b^	217	0.96	0.92–0.99	0.017	180	0.98	0.93–1.03	0.462

Abbreviations: AME, Aide médicale de l'Etat; CI, confidence interval; CMU, couverture maladie universelle; FGM, female genital mutilation; OR, odds ratio.

^a^ For the multivariable model, only variables statistically significant in univariable analyses (*p* < 0.05) were entered. Therefore, the final model was adjusted for seven variables. The model included 180 complete cases. Two variables were not retained in the multivariable model. “Recent immigration” was not included because of sparse data (cell counts <5). The “socio‐professional category” had a variance inflation factor (VIF) of 5, suggesting substantial collinearity with at least one of the remaining covariates.

^b^ Per one‐year increase in age (continuous variable).

In the Bayesian multivariable logistic regression, the strongest evidence for non‐progression to surgery was observed for lack of support within the care pathway (OR 3.38, 95% credible interval [CrI] 1.49–7.98; posterior probability 99.9%) and financial vulnerability (support from relatives/community: OR 5.66, 95% CrI 1.27–24.29; posterior probability 99.0%; state welfare benefits: OR 3.34, 95% CrI 1.27–9.10; posterior probability 99.2%). Strong evidence was also observed for memory of the FGM event (OR 1.87, 95% CrI 0.88–4.09; posterior probability 94.9%) (Table 3). Results were robust to prior specification in sensitivity analyses using alternative weakly informative priors (Figure 1).

**TABLE 3:** Bayesian multivariable logistic regression.

Variable^a^	OR (95% CrI)	*P* (direction)	Direction of effect	Evidence level
Financial resources—Support from relatives/community	5.66 (1.27–24.29)	99.0%	Risk ↑	**Very strong**
Financial resources—State welfare benefits	3.34 (1.27–9.10)	99.2%	Risk ↑	**Very strong**
Financial resources—Charity	0.61 (0.08–3.75)	70.2%	Protective ↓	Weak
Health insurance—CMU	0.79 (0.29–2.06)	68.5%	Protective ↓	Weak
Health insurance—AME	0.64 (0.19–2.01)	77.3%	Protective ↓	Moderate
Health insurance—None	1.29 (0.28–6.01)	62.7%	Risk ↑	Weak
Support for care pathway—No	3.38 (1.49–7.98)	99.9%	Risk ↑	**Very strong**
Marital status—Cohabiting	1.73 (0.71–4.10)	89.2%	Risk ↑	Moderate
Marital status—Divorced	0.44 (0.10–1.78)	87.3%	Protective ↓	Moderate
Marital status—Single	0.64 (0.19–2.08)	77.5%	Protective ↓	Moderate
Memory of FGM event—Yes	1.87 (0.88–4.09)	94.9%	Risk ↑	**Strong**
Reported dyspareunia—Yes	1.13 (0.46–2.86)	60.7%	Risk ↑	Weak
Age at initial medical care	0.98 (0.93–1.03)	81.2%	Protective ↓	Moderate

Abbreviations: AME, Aide médicale de l'Etat; CI, confidence interval; CMU, couverture maladie universelle; FGM, female genital mutilation; OR, odds ratio; *P*(direction), posterior probability that the effect is in the reported direction.

^a^ Variables are listed in the order used in the multivariable logistic regression model. Posterior odds ratios with 95% credible intervals (CrI), directional probabilities, and evidence levels (reference prior: *N*(0, 2.5); Markov Chain Monte Carlo (MCMC): 8 chains × 4000 iterations). Protective effects are indicated as *Protective ↓* when OR <1 and risk effects as *Risk ↑* when OR >1. Evidence level: very strong, >95%; strong, 90%–95%; moderate, 75%–89%; weak, <75%. Estimates are based on complete‐case analyses (*n* = 180) adjusted for all seven variables (financial resources, health insurance coverage, support for care pathway, marital status, memory of FGM event, reported dyspareunia, and age at initial medical care).

**FIGURE 1:**
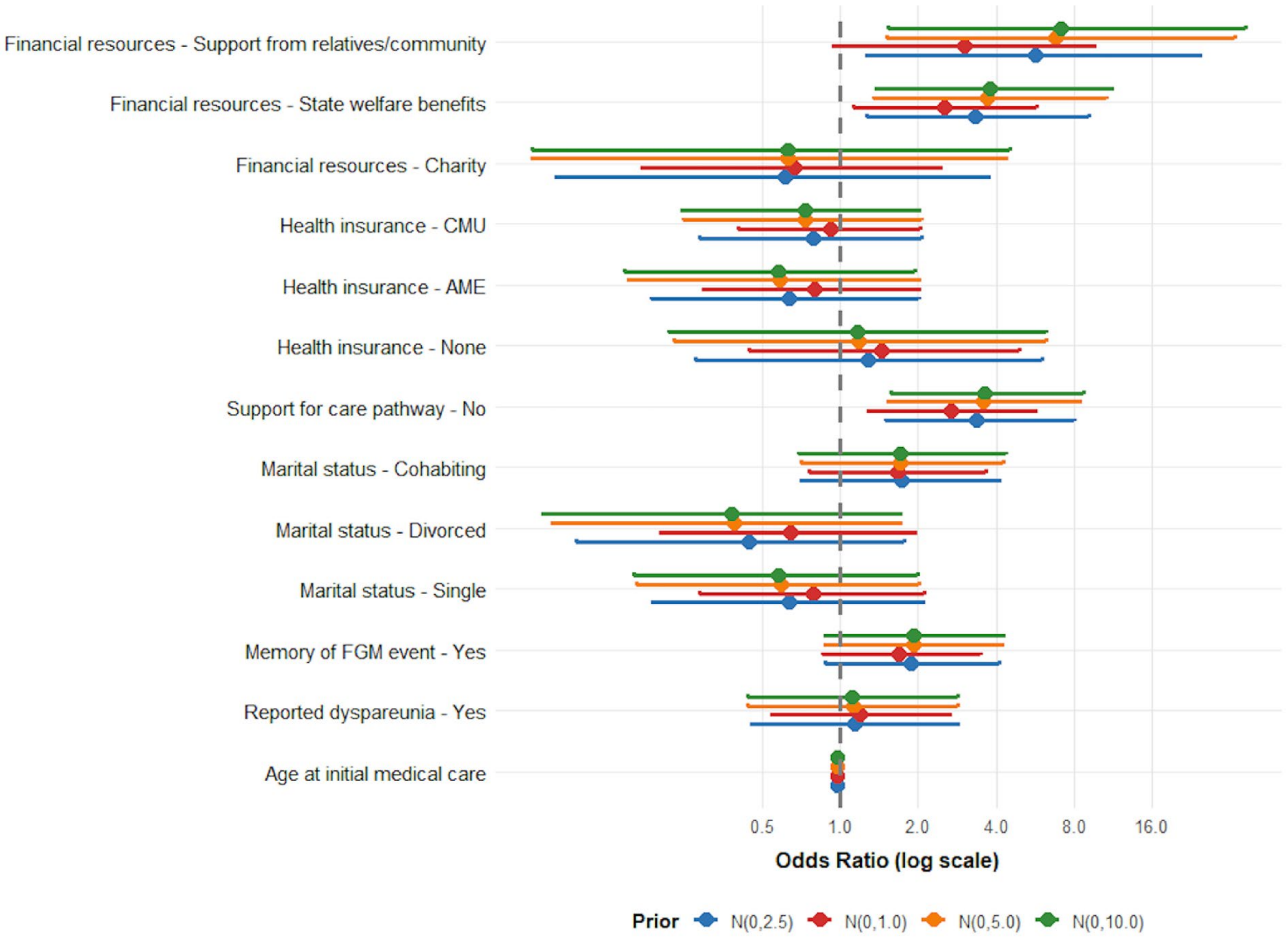
Prior sensitivity analysis for the Bayesian model. Posterior odds ratios (points) and 95% credible intervals (lines). Posterior estimates were similar across alternative weakly informative priors; results are reported using the reference prior *N*(0, 2.5), commonly used in the literature. AME, Aide médicale de l’Etat; CMU, couverture maladie universelle; FGM, female genital mutilation.

## 4 DISCUSSION

### 4.1 Main findings

In this cohort of 220 women enrolled in a reconstructive care pathway after FGM, 35% did not proceed to surgery. Across analyses, lack of support within the care pathway and socioeconomic vulnerability emerged as the main determinants of non‐progression. In the adjusted model, women without support within the care pathway (OR 3.28, 95% CI 1.46–7.55) and those relying on state welfare benefits (OR 3.43, 95% CI 1.28–9.80) or financial support from relatives/community (OR 6.18, 95% CI 1.40–28.9) had substantially higher risk of not proceeding to surgery. Model discrimination was good (AUC 0.75). Bayesian analyses confirmed these findings, providing very strong evidence for these socioeconomic factors and strong evidence for the role of memory of the FGM event in non‐progression to surgery (OR 1.87, 95% CrI 0.88–4.09).

### 4.2 Strengths and limitations

This study has several strengths. First, to our knowledge, it is the first study specifically designed to investigate factors associated with discontinuation of a clitoral reconstructive surgical pathway after FGM. By focusing not only on women who ultimately underwent surgery but also on those who did not proceed despite initial engagement in care, it addresses a major gap in the literature and provides a more complete understanding of women's trajectories after seeking reconstructive care. Second, The combination of frequentist and Bayesian analyses strengthened the robustness of the findings and provided complementary information on the probability and direction of associations, allowing a more nuanced interpretation of uncertainty in the context of a relatively modest sample size. Third, the study was conducted in a specialized referral center offering integrated medico‐psycho‐social care, ensuring a standardized pathway and detailed clinical information on women's social, medical, and psychosocial characteristics. However, several limitations should be considered. First, the retrospective monocentric design might limit the generalizability of the findings to other healthcare settings or countries with different healthcare systems and social contexts. Second, some variables were subject to missing data, and multivariable analyses were, therefore, restricted to complete cases. Although the similar proportion of non‐progression among included and excluded women suggests limited selection bias, this restriction might have reduced statistical power to detect associations. Third, certain potentially relevant determinants, such as language barriers, were not systematically recorded in the medical files and could not be analyzed. Finally, the observational design does not allow causal inference, and the associations observed should be interpreted as indicators of underlying social and care‐related mechanisms rather than direct causal effects.

### 4.3 Interpretation

Our findings extend current knowledge by empirically demonstrating how social support and socioeconomic conditions influence women's trajectories within reconstructive care pathways after FGM. These results align with international recommendations emphasizing that FGM management should rely on a comprehensive, multidisciplinary, and culturally sensitive approach integrating medical, psychological, sexual, and social dimensions.^1^, ^21^ The WHO framework specifically stresses that reconstructive surgery should be offered within specialized teams capable of addressing psychological trauma, sexual health, and social vulnerability while supporting informed decision‐making and long‐term care. Consistent with our findings, WHO systematic reviews further highlight that outcomes after FGM are strongly shaped by social and structural determinants such as migration history, education, and access to health insurance.^22^ Moreover, similar risk factors for the recurrence of violence are observed among women without independent income and experiencing socioeconomic vulnerabilities. The WHO promotes women's economic empowerment as a key strategy to strengthen autonomy and reduce intimate partner violence.^23^, ^24^

A growing body of literature supports this multidimensional perspective. FGM is associated with a wide range of obstetric, sexual, and psychological complications that may be exacerbated by limited access to specialized care.^25^, ^26^ It can impair multiple domains of sexual function and wellbeing, reinforcing the need for integrated psychosexual and medical support.^27^, ^28^ Importantly, psychological trauma and chronic pain may persist independently of the anatomical type of mutilation, emphasizing that the consequences of FGM cannot be reduced to physical damage alone.^7^, ^29^

Beyond clinical sequelae, access to reconstructive surgery appears to be strongly influenced by women's social position and degree of integration in the host society. Women who undergo reconstructive surgery are often better informed, financially stable, and socially supported, whereas recently arrived or socioeconomically vulnerable women remain underrepresented in surgical cohorts.^30^, ^31^ A qualitative review highlights that women affected by FGM often face multiple barriers to care, including language difficulties, lack of information, family constraints, and “hit‐and‐miss” service organization, all of which may limit access to and continuity within specialized pathways.^31^ As a result, published surgical cohorts might include relatively selected populations with more favorable social conditions, potentially leading to an overestimation of the functional benefits observed after surgery.^32^, ^33^ In contrast, in our study, the rate of discontinuation was lower, and the proportion of women who ultimately underwent reconstructive surgery was higher than that reported in the literature.^11^, ^15^ These findings might reflect the added value of the specialized multidisciplinary care pathway implemented in our setting, which provides individualized support to address women's vulnerabilities and helps them navigate decision‐making beyond psychosocial and economic factors alone. In this context, the strong association observed between lack of support within the care pathway and non‐progression to surgery highlights the central role of accompaniment and continuity of care in complex treatment trajectories. Indeed, multidisciplinary pathways combining gynecological, psychological, and social support have been shown to improve continuity of care and to better adapt treatment plans to women's expectations and needs.^10^ Our findings suggest that sustained support throughout the care pathway might be critical in enabling women to proceed to surgery when this option aligns with their wishes.

Importantly, the Bayesian analysis confirmed the associations observed in the frequentist models, providing very strong evidence for the role of socioeconomic vulnerability and lack of support in non‐progression to surgery. It also revealed that memory of the FGM event plays a role in non‐progression, which was not fully captured in the frequentist analysis or in routinely collected data, likely because its effect was masked by its correlation with income. This interpretation is consistent with the patient interviews, in which recollection of the event emerged as an important element in women's narratives around surgery. Taken together, these results suggest that reconstructive surgery after FGM should not be considered solely as a surgical intervention but rather as one component of a broader care trajectory shaped by migration history, social integration, psychological readiness, and the availability of supportive multidisciplinary care.

## 5 CONCLUSION

Access to clitoral reconstructive surgery after FGM appears to depend largely on social support and socioeconomic stability rather than on clinical factors alone. Women facing financial vulnerability or lacking support within the care pathway were less likely to c, highlighting persistent inequalities in access to specialized care. Identifying these risk factors for non‐progression might help clinicians identify women at higher risk of discontinuing the pathway and provide earlier, tailored support to address these vulnerabilities. This underlines the need for reconstructive surgery to be delivered within coordinated multidisciplinary pathways that address not only surgical needs but also psychological, sexual, and social dimensions of care. Future research should further explore women's trajectories and decision‐making processes to inform more equitable, patient‐centered models of care.

## AUTHOR CONTRIBUTIONS

Maëlle Silvestre De Sacy (MSC), Sarah Abramowicz (SA), Félicia Joinau‐Zoulovits (FJZ) had full access to all the data in the study and take responsibility for the integrity of the data and the accuracy of the data analysis. Study concept and design: MSC, SA, FJZ. Acquisition of data: MSC. Analysis and interpretation of data: FJZ, SA. Drafting of the manuscript: SA. Critical revision of the manuscript for important intellectual content: SA, BR. Statistical analysis: FJZ. Study supervision: FJZ, SA.

## FUNDING INFORMATION

None.

## CONFLICT OF INTEREST STATEMENT

None.

## ETHICS STATEMENT

The study complied with French CNIL‐MR004 and GDPR data protection regulations and accepted by the CEROG committee in 2024 under the reference #2024‐GYN‐0707.

## PATIENT CONSENT STATEMENT

The study complied with French CNIL‐MR004 and GDPR data protection regulations and all participants were informed and did not object to the reuse of their data in this study.

## Data Availability

The anonymized dataset supporting the findings of this study is available from the corresponding author upon reasonable request.

## References

[1] World Health Organization . WHO Guideline on the Prevention of Female Genital Mutilation and Clinical Management of Complications. World Health Organization; 2025. Licence: CC BY‐NC‐SA 3.0 IGO. ISBN 978‐92‐4‐010728‐1. 40397770

[2] World Health Organisation . Eliminating female genital mutilation: an interagency statement—OHCHR, UNAIDS, UNDP, UNECA, UNESCO, UNFPA, UNHCR, UNICEF, UNIFEM, WHO. World Health Organization; 2008.

[3] Utz‐Billing I , Kentenich H . Female genital mutilation: an injury, physical and mental harm. J Psychosom Obstet Gynecol. 2008;29:225‐229. 10.1080/01674820802547087 19065392

[4] Byard RW , Behnia‐Willison F . Female genital mutilation – an overview for forensic practitioners. J Forensic Leg Med. 2024;101:102624. 10.1016/j.jflm.2023.102624 38011766

[5] Morison L , Scherf C , Ekpo G , et al. The long‐term reproductive health consequences of female genital cutting in rural Gambia: a community‐based survey. Trop Med Int Health. 2001;6:643‐653. 10.1046/j.1365-3156.2001.00749.x 11555430

[6] Berg RC , Underland V . Gynecological Consequences of Female Genital Mutilation/Cutting (FGM/C). Knowledge Centre for the Health Services at The Norwegian Institute of Public Health (NIPH); 2014. 29320038

[7] Behrendt A , Moritz S . Posttraumatic stress disorder and memory problems after female genital mutilation. Am J Psychiatry. 2005;162:1000‐1002. 10.1176/appi.ajp.162.5.1000 15863806

[8] Berg RC , Denison E , Fretheim A . Psychological, Social and Sexual Consequences of Female Genital Mutilation/Cutting (FGM/C): A Systematic Review of Quantitative Studies. Knowledge Centre for the Health Services at The Norwegian Institute of Public Health (NIPH); 2010. 29320049

[9] WHO Study Group on Female Genital Mutilation and Obstetric Outcome . Female genital mutilation and obstetric outcome: WHO collaborative prospective study in six African countries. Lancet. 2006;367:1835‐1841. 10.1016/S0140-6736(06)68805-3 16753486

[10] Antonetti Ndiaye E , Fall S , Beltran L . Intérêt de la prise en charge pluridisciplinaire des femmes excisées. J Gynecol Obstet Biol Reprod. 2015;44:862‐869. 10.1016/j.jgyn.2015.01.008 25721349

[11] Merckelbagh HM , Nicolas MN , Piketty MP , Benifla JL . Évaluation d'une prise en charge multidisciplinaire chez 169 patientes excisées demandeuses d'une chirurgie réparatrice. Gynecol Obstet Fertil. 2015;43:633‐639. 10.1016/j.gyobfe.2015.09.002 26439872

[12] Lesclingand M , Andro A , Lombart T . Estimation du nombre de femmes adultes ayant subi une mutilation génitale féminine vivant en France. Bull Epidemiol Hebd. 2019;21:392‐399.

[13] Foldes P . Reconstructive plastic surgery of the clitoris after sexual mutilation. Prog Urol. 2004;14:47‐50. 15098751

[14] Chevrot A , Lousquy R , Arfi A , Haddad B , Paniel BJ , Touboul C . Technique opératoire: la transposition clitoridienne. J Gynecol Obstet Biol Reprod. 2015;44:787‐791. 10.1016/j.jgyn.2015.02.013 25818112

[15] Foldès P , Cuzin B , Andro A . Reconstructive surgery after female genital mutilation: a prospective cohort study. Lancet. 2012;380:134‐141. 10.1016/S0140-6736(12)60400-0 22695031

[16] Abdulcadir J , Rodriguez MI , Say L . A systematic review of the evidence on clitoral reconstruction after female genital mutilation/cutting. Int J Gynecol Obstet. 2015;129:93‐97. 10.1016/j.ijgo.2014.11.008 PMC6434902 25638712

[17] Foldes P , Louis‐Sylvestre C . Résultats de la réparation chirurgicale du clitoris après mutilation sexuelle: 453 cas. Gynecol Obstet Fertil. 2006;34:1137‐1141. 10.1016/j.gyobfe.2006.09.026 17137821

[18] Abramowicz S , Oden S , Dietrich G , Marpeau L , Resch B . Évaluation des résultats anatomiques, fonctionnels et identitaires après transposition du clitoris chez 30 patientes. J Gynecol Obstet Biol Reprod. 2016;45:963‐971. 10.1016/j.jgyn.2016.03.010 27118679

[19] Lesclingand M , Sylla F , Andro A , Boisson S . Projet MSF‐PREVAL. Rapport final. Ministère chargé de l'égalité entre les femmes et les hommes, de la diversité et de l'égalité des chances. 2022.

[20] Dabi Y , Thubert T , Fuchs F , Barjat T , Belaisch‐Allart J , Ceccaldi PF . How is functioning the ethical review board « Comité d'Ethique pour La Recherche En Obstétrique et Gynécologie » (CEROG)? J Gynecol Obstet Hum Reprod. 2022;51:102352. 10.1016/j.jogoh.2022.102352 35247608

[21] Haute Autorité de Santé (HAS) . Prise en charge des mutilations sexuelles féminines par les professionnels de santé de premier recours. Recommandation de Bonne Pratique. 2020.

[22] World Health Organization . Systematic Reviews to Inform the Update of the World Health Organization's Guidelines on Female Genital Mutilation. Request for Proposals No. 2022/HQ/UHL/SRH/AVP/01. World Health Organization; 2022.

[23] Kim JC , Watts CH , Hargreaves JR , et al. Understanding the impact of a microfinance‐based intervention on Women's empowerment and the reduction of intimate partner violence in South Africa. Am J Public Health. 2007;97:1794‐1802. 10.2105/AJPH.2006.095521 PMC1994170 17761566

[24] World Health Organization . Violence against Women: a Priority Health Issue. World Health Organization; 1997.

[25] Pallitto C , Ruiz‐Vallejo F , Mochache V , Stein K , Vogel JP , Petzold M . Exploring the health complications of female genital mutilation through a systematic review and meta‐analysis. BMC Public Health. 2025;25:1387. 10.1186/s12889-025-21584-z PMC11995580 40229755

[26] Sylla F , Moreau C , Andro A . A systematic review and meta‐analysis of the consequences of female genital mutilation on maternal and perinatal health outcomes in European and African countries. BMJ Glob Health. 2020;5:e003307. 10.1136/bmjgh-2020-003307 PMC7780522 33380410

[27] Pérez‐López FR , Ornat L , López‐Baena MT , Pérez‐Roncero GR , Tajada‐Duaso MC , Chedraui P . Association of female genital mutilation and female sexual dysfunction: a systematic review and meta‐analysis. Eur J Obstet Gynecol Reprod Biol. 2020;254:236‐244. 10.1016/j.ejogrb.2020.09.031 33011507

[28] Buggio L , Facchin F , Chiappa L , Barbara G , Brambilla M , Vercellini P . Psychosexual consequences of female genital mutilation and the impact of reconstructive surgery: a narrative review. Health Equity. 2019;3:36‐46. 10.1089/heq.2018.0036 PMC6386073 30805570

[29] Bazzoun Y , Aerts L , Abdulcadir J . Chronic vulvar pain after female genital mutilation/cutting: a retrospective study. Sex Med. 2021;9:100425. 10.1016/j.esxm.2021.100425 PMC8498950 34520933

[30] Berg RC , Taraldsen S , Said MA , Sørbye IK , Vangen S . Reasons for and experiences with surgical interventions for female genital mutilation/cutting (FGM/C): a systematic review. J Sex Med. 2017;14:977‐990. 10.1016/j.jsxm.2017.05.016 28666656

[31] Evans C , Tweheyo R , McGarry J , et al. Seeking culturally safe care: a qualitative systematic review of the healthcare experiences of women and girls who have undergone female genital mutilation/cutting. BMJ Open. 2019;9:e027452. 10.1136/bmjopen-2018-027452 PMC6549627 31147364

[32] Auricchio V , Garzon S , Pomini P , et al. Clitoral reconstructive surgery after female genital mutilation: a systematic review. Sex Reprod Healthc. 2021;29:100619. 10.1016/j.srhc.2021.100619 33845447

[33] Gnofam M , Crequit S , Renevier B , Abramowicz S . Prognostic factors of poor surgical outcome after clitoral reconstruction in women with female genital mutilation/cutting. J Sex Med. 2023;21:59‐66. 10.1093/jsxmed/qdad150 38014807

